# Supplementary material for: Multi-omics analyses reveal that the gut microbiome and its metabolites promote milk fat synthesis in Zhongdian yak cows
Source: PeerJ. 2022 Dec 2;10:e14444. doi: 10.7717/peerj.14444 (PMC9744170; doi:10.7717/peerj.14444)
Supplement: Supplemental Information 13 [file peerj-10-14444-s013.zip › Web_Report/Metabolites_annotation/Lipidmaps/meta_lipidmaps_anno.pdf]

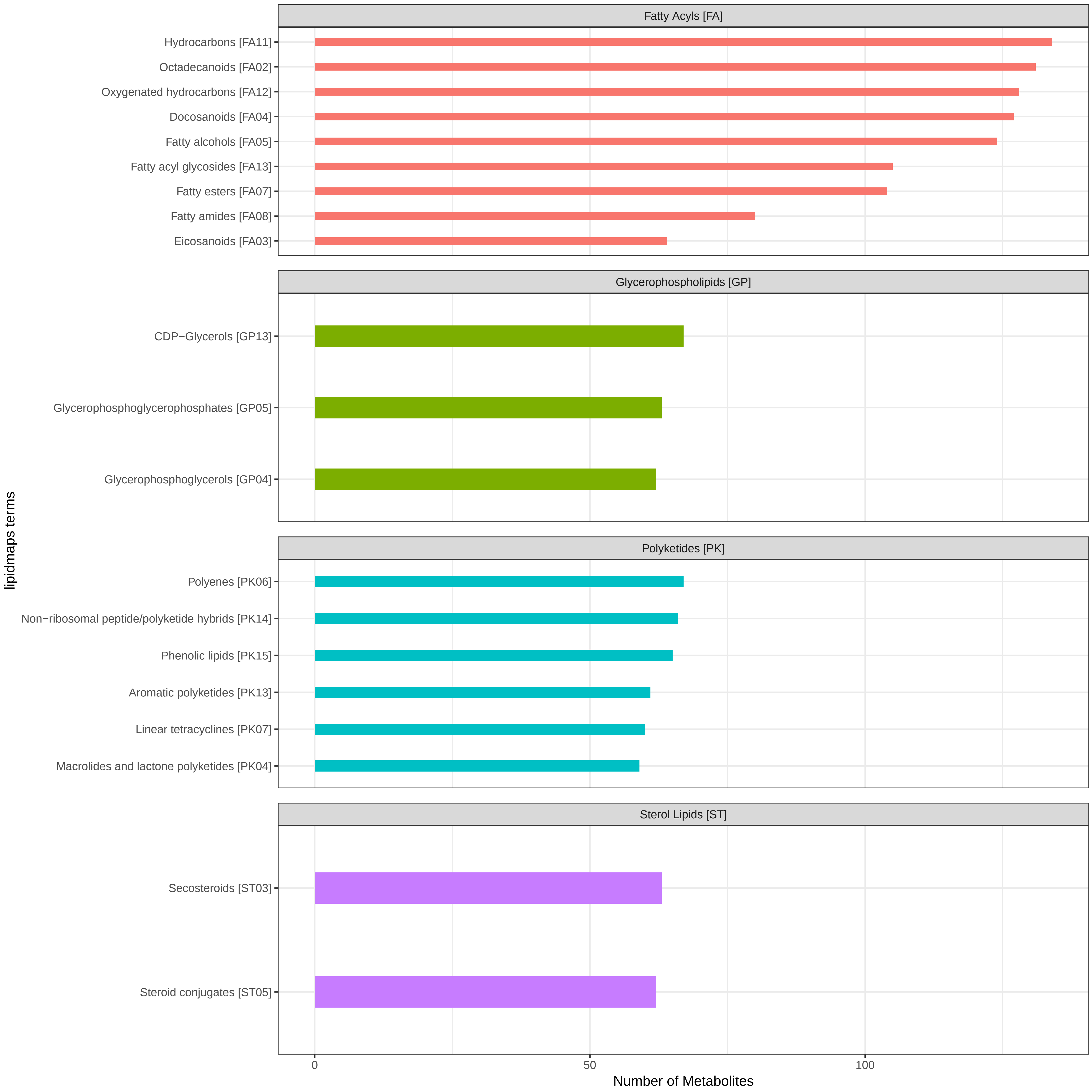

Fatty Acyls [FA]
Hydrocarbons [FA11]
Octadecanoids [FA02]
Oxygenated hydrocarbons [FA12]
Docosanoids [FA04]
Fatty alcohols [FA05]
Fatty acyl glycosides [FA13]
Fatty esters [FA07]
Fatty amides [FA08]
Eicosanoids [FA03]
Glycerophospholipids [GP]
CDP−Glycerols [GP13]
Glycerophosphoglycerophosphates [GP05]
Glycerophosphoglycerols [GP04]
Polyketides [PK]
Polyenes [PK06]
Non−ribosomal peptide/polyketide hybrids [PK14]
Phenolic lipids [PK15]
Aromatic polyketides [PK13]
Linear tetracyclines [PK07]
Macrolides and lactone polyketides [PK04]
Sterol Lipids [ST]
Secosteroids [ST03]
Steroid conjugates [ST05]
lipidmaps terms
0
50
100
Number of Metabolites
